# Performances of the PIPER scalable child human body model in accident reconstruction

**DOI:** 10.1371/journal.pone.0187916

**Published:** 2017-11-14

**Authors:** Chiara Giordano, Xiaogai Li, Svein Kleiven

**Affiliations:** Division of Neuronic Engineering, School of Technology and Health, KTH Royal Institute of Technology, Stockholm, Sweden; Beihang University, CHINA

## Abstract

Human body models (HBMs) have the potential to provide significant insights into the pediatric response to impact. This study describes a scalable/posable approach to perform child accident reconstructions using the Position and Personalize Advanced Human Body Models for Injury Prediction (PIPER) scalable child HBM of different ages and in different positions obtained by the PIPER tool. Overall, the PIPER scalable child HBM managed reasonably well to predict the injury severity and location of the children involved in real-life crash scenarios documented in the medical records. The developed methodology and workflow is essential for future work to determine child injury tolerances based on the full Child Advanced Safety Project for European Roads (CASPER) accident reconstruction database. With the workflow presented in this study, the open-source PIPER scalable HBM combined with the PIPER tool is also foreseen to have implications for improved safety designs for a better protection of children in traffic accidents.

## Introduction

The protection of children in motor vehicle crashes has improved thanks to the introduction of child restraint systems (CRSs). Nevertheless, children car occupants up to 14 years of age are involved in 32% of European road traffic fatalities [1] and car crashes remain the second leading cause of injury for children between 5 and 14 years old (y.o.) [2].

Traditionally, occupant safety is evaluated by crash tests using Anthropometric Test Devices (ATDs). For children, Q-dummies are in use or considered in regulation R129 and consumer testing (Euro NCAP, ADAC), which include a deformable spine and rib cage to allow realistic flexion, extension and lateral flexion rotational behavior. However, the biofidelity of Q-dummies has been questioned in several studies [3–5], especially their capability to provide detailed injury responses [3, 6]. Simplifications and limitations in dummies indeed arise from the need of a physical implementation. Also, dummies are typically designed to match regulation requirements (R44, R129 in Europe) with performance targeting mostly at kinematics behaviors.

Human Body Models (HBMs), and in particular Finite Element (FE) models, have the potential to provide significant insights into the pediatric response to impact. The use of HBMs enables assessment of local mechanical behaviors and estimation of human tolerances (injury risk curves) to external forces. Unlike dummies, which are only available in few dimensions and ages, HBMs can be scaled to multiple dimensions [7] and responses can be evaluated in multiple directions (omnidirectionality). Also, FE models can accurately represent the complex anatomy of the human body and the growth with age [7]. However, while great effort has been spent to generate full body models for adults [8, 9], the availability of child human models has been more limited and based on numerous assumptions [10–12]. Furthermore, the data available to verify the response of child models is scarcer than for adults.

**Fig 1:**
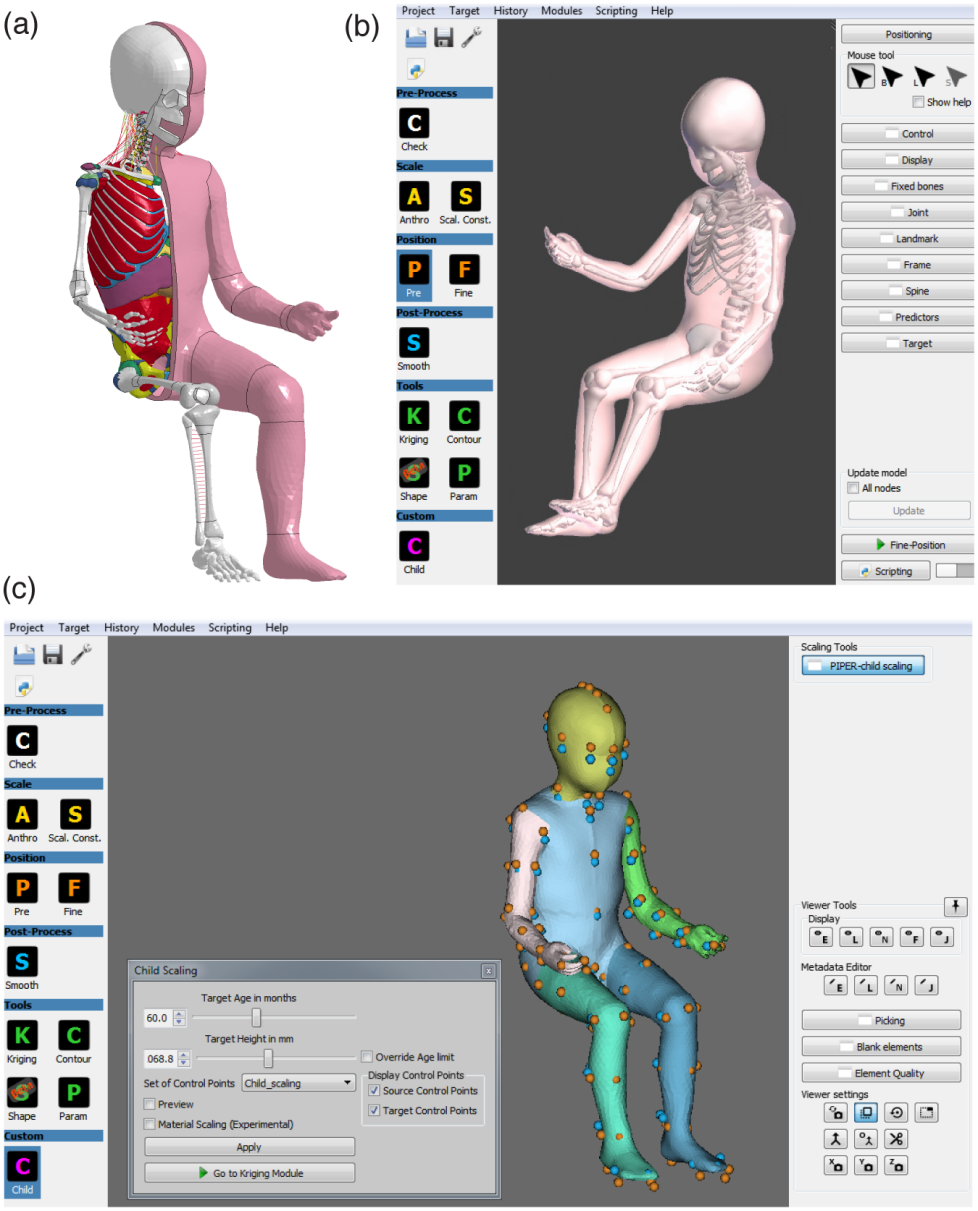
Overview of the PIPER scalable HBM. (a) Model with skeleton and internal organs exposed; (b) Positioning of the HBM with the PIPER tool; (c) Personalization (scaling) of the HBM with the PIPER tool.

In 2016, Beillas et al. [13] presented the development of a detailed child full body model continuously scalable between 1.5 and 6 years of age accounting the growth process (Fig 1a). The PIPER scalable child HBM was compared to experimental references for all body regions and the results were very encouraging with generally a good match between the model response and the reference [13]. The unicity of the PIPER scalable child HBM is the integration/compatibility of the model into the PIPER software framework. The PIPER tool was developed under the PIPER project and allows positioning and personalization of HBMs (Fig 1b and 1c). This continuously scalable approach [7, 13] enables to perform computer simulations with HBMs of different ages, different sizes for a given age, or with HBMs in different positions, all starting from a single mesh connectivity.

The objective of this study is to use the PIPER scalable HBM in combination with the PIPER tool for performing a series of child accident reconstructions. The dynamic performances of the HBM will be examined in real-life crash scenarios and compared to the accident reconstruction capability of the Q-dummies. This investigation is therefore a first attempt to determine child injury tolerances based on scalable-posable HBMs.

## Materials and methods

### Accident reconstructions

Three full-scale accident reconstructions were selected for this study based on real accident data collected during the CASPER project [3]. Accident data included reports on injury location, injury severity, restraint conditions and in-depth investigation of cars.

Case 2012 involved a 26 months old (m.o.) female child occupant suffering Maximum Abbreviated Injury Scale (MAIS) 4 injuries. The child was sitting on the rear right seat of a Renault Megan Scenic II that, because of wet road, impacted frontally a BMW 525 tds. The child sustained a hard impact of the head with the front seatback resulting in, among other injuries, a fracture at the roof of the left orbit, brain contusion and extra dural hematoma.

Case 2017 was a fronto-lateral collision between a Renault Megan Scenic II and a Citroen Xsara due to a hazardous maneuver. It involved a 5 y.o. male child occupant suffering no injuries (MAIS 0). The child was sitting on a low-back booster cushion Team Tex E2 03 6018 and was properly belted.

Case 2043 involved a 5 y.o. female child occupant suffering MAIS 6 injuries. A Honda Jazz lost control on a rural street and collided frontally with a tree. No signs of breaking were visible. The child sustained extensive severe injuries, such as diffuse axonal head trauma, dislocation of the cervical spine at the level of the 3rd Cervical Vertebra (C3), lung contusion, and laceration of liver, spleen and left kidney.

For the above three cases, besides the detailed accident data collected, physical accident reconstructions were also performed in crash-test laboratories in the CASPER project, using similar vehicles, CRS and child dummies of a size as close as possible to the children involved in the accident [3]. Sensor readings from the physical accident reconstructions, such as pillar accelerations of the vehicle, and dummy sensor readings were available from the accident reproduction.

These accidents were selected and reconstructed in this study based on representativeness for the PIPER scalable HBM (children between 1.5 and 6 y.o.) and reproducibility with FE model (detailed accident reconstruction report). Further details about the real accidents and child occupant injuries can be found in the supporting documents S1, S2 and S3 Files. More information about the physical reconstructions using Q-dummies is found in [3].

### Environment FE models

**Fig 2:**
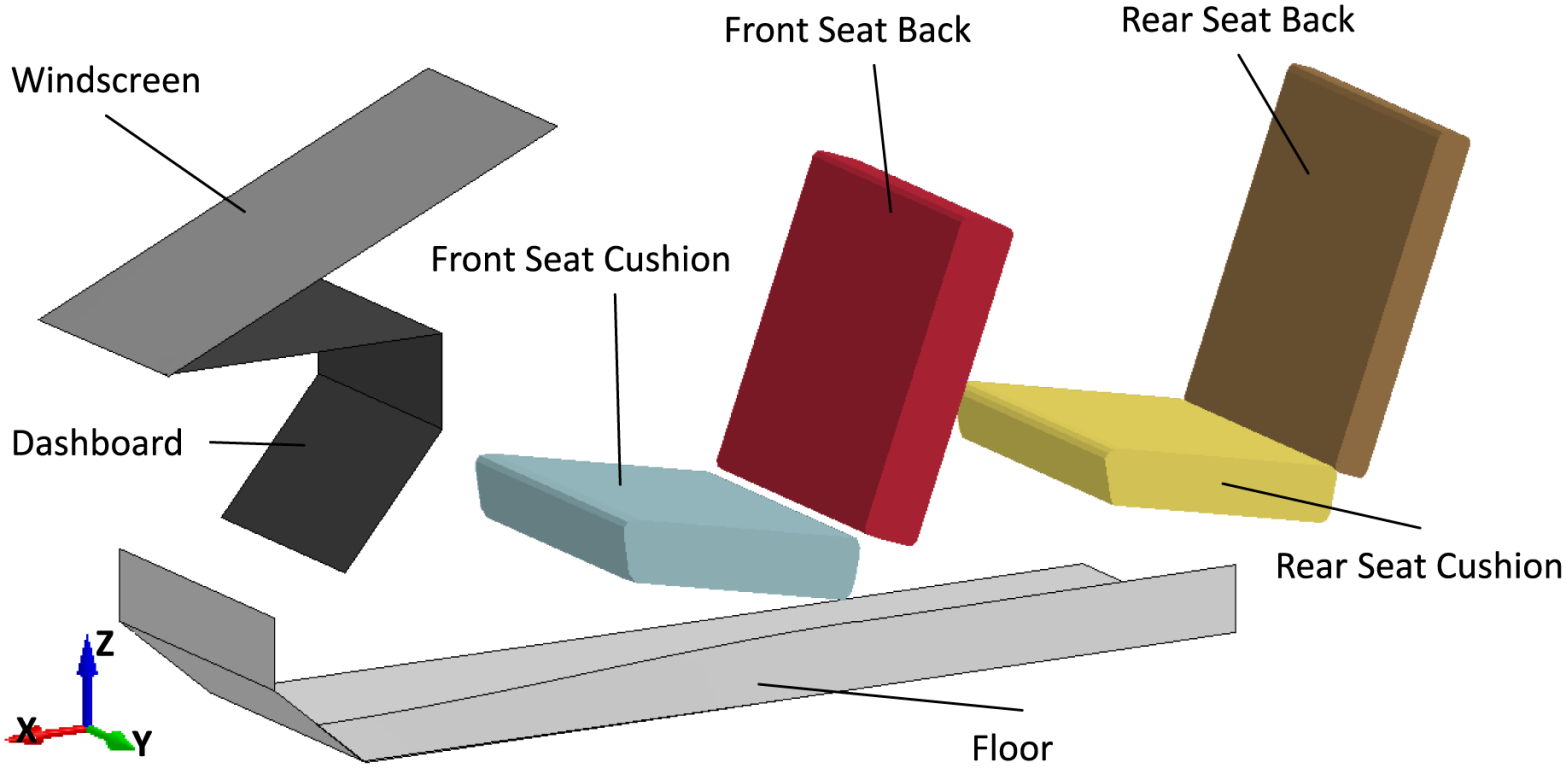
The PIPER generalized car environment model v1.0. This model consists of all relevant components of a generic vehicle for use in impact simulations.

The FE simulations were performed using the PIPER generalized car environment model v1.0 (Fig 2). This model consists of all relevant components of a generic vehicle for use in impact simulations and is parameterized in terms of component positions and angles. Parameters of the model are, for example, seat dimensions, seat inclinations and seatbelt positions. Validation of the car environment model included comparison to a physical accident reconstruction from the CASPER database (CCN 0391) and seat cushion intrusion tests at the rear bench [14]. In the current study, the generic model was adjusted to describe the specific interiors of a Renault Megan Scenic II for Case 2012 and 2017 and the interior of a Honda Jazz for Case 2043. Reports on restraint conditions and in-depth investigation of cars were used to produce environments as close as possible to the vehicles actually involved in the accidents. Table 1 reports the parameter values used to reproduce the vehicles. Furthermore, for Case 2012, besides the global parametric adjustments, the mesh of the front seat was modified to reflect its real geometry with the purpose to better model the head interaction with the car seat (the severe head impact occurred onto a metal bar located under the foam covering of the front seat). Similarly for Case 2017, the mesh of the back seat was modified to reflect its real geometry.

**Table 1:** Values assigned to the parametric environment model to reproduce the vehicles of Case 2012, 2017 and 2043. Only the rear and front seat were used in simulations.

Parameter Name	Parameter Value		
	Case 2012	Case 2017	Case 2043
RSC Scaling X	0.8234	0.8234	0.9334
RSC Scaling Y	0.6820	0.6820	0.6820
RSC Scaling Z	0.8010	0.8010	0.8030
RSC Rotation Y	-10°	-10°	-4°
RSB Scaling X	1.1000	1.1000	1.1000
RSB Scaling Y	0.6820	0.6820	0.6820
RSB Scaling Z	1.1003	1.1003	1.1003
RSB Rotation Y	-14°	-14°	-14°
FSC Scaling X	1.0000	1.0000	1.0000
FSC Scaling Y	0.9254	0.9254	0.9254
FSC Scaling Z	1.1050	1.1050	1.1050
FSC Rotation Y	-10°	-10°	-10°
FSB Scaling X	1.0050	1.0050	1.0050
FSB Scaling Y	0.9254	0.9254	0.9254
FSB Scaling Z	1.0003	1.0003	1.0003
FSB Rotation Y	-9°	-14°	-14°

RSC – Rear Seat Cushion

RSB – Rear Seat Back

FSC – Front Seat Cushion

FSB – Front Seat Back

**Fig 3:**
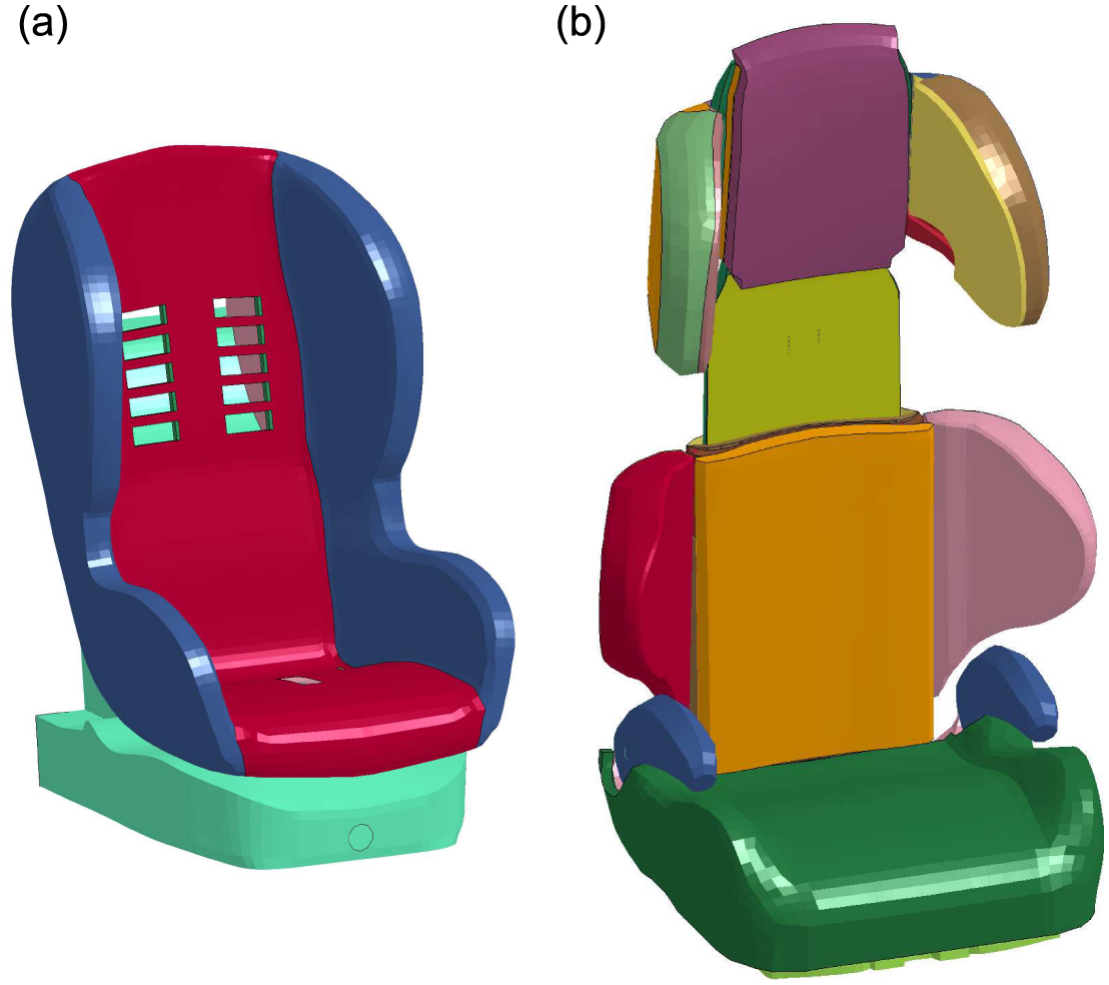
CRS models used for the FE simulations. (a) CRS model for children between 9-18 Kg or of age between 9 months and 4 years; (b) CRS model for children between 15-25 Kg or of age between 4 and 6 years.

The CRS models used for the FE simulations were developed during the CASPER project [15] (Fig 3a and 3b). The CRS model for group 1 (children between 9-18 Kg – 9 m.o. to 4 y.o.) had a design based on the Maxi Cosi Priori Fix and was modelled with an elastic seat shell and a rigid seat base. The seat shell was covered with a foam covering. Validation of the model included comparison to three physical tests performed with Q3 dummies [15]. In the current study the generic model had a configuration very similar to the CRS described in Case 2012. Thus, the generic model was used without adjusting dimensions and the seat was reclined of -8 degrees with respect to the vertical.

The CRS model for group 2 (children between 15-25 Kg – 4 y.o. to 6 y.o.) had a design based on the Jane Indy Racing seat (Fig 3b) with movable wings in the head/chest areas and a displaceable backrest to customize it to the size of the child. Validation of the CSR model included comparison to sled test results with a Q6 dummy and interpolation to represent several CRSs [15]. In the current study the generic CRS model was adjusted to describe the specific interiors for Case 2017 and Case 2043. Table 2 reports the parameters used in the simulations to adjust the model.

**Table 2:** Values assigned to the parametric CRS model (group 2) to reproduce the CRS of Case 2017 and 2043. Case 2017 involved a child sitting on a low back booster. Therefore, backrest height and rotation values are not applicable (N/A).

Parameter Name	Parameter Value	
	Case 2017	Case 2043
Backrest Height	N/A	629 mm
Backrest Rotation	N/A	-16°
Side Wall Rotation	N/A	0°
Side Protection Rotation	0°	0°
CRS Rotation Y	-10°	4°

### PIPER scalable HBM

The PIPER scalable HBM (Fig 1a) is a detailed child full body model able to describe the growth process and the variation in relevant anatomical regions for children between 1.5 and 6 y.o. The baseline model describes the anatomy of an average 6 y.o. child and has a total mass of 23 Kg. The anthropometric dimensions were normalized by nonlinear scaling using Generator of Body Data (GEBOD) [16] regressions as reference. Overall, the model is composed of approximately 542,000 elements distributed into more than 350 parts describing the main anatomical structures. The head, neck and lower extremities are meshed with hexahedral elements, while the flesh and trunk are meshed with tetrahedral elements. The model was developed in the LS-Dyna explicit FE code and has a time step of 0.32 μs obtained with marginal mass scaling (15 grams added). The PIPER scalable child HBM was compared to experimental references for all body regions and the results were very encouraging with generally a good match between the model response and the reference. The validation matrix included drop and compression tests for the head, bending and tensile tests for the cervical spine, pendulum and belt interaction tests for the trunk, bending tests for the lower extremities and full body sled tests for the mobility of the spine. Shoulder and pelvis performances were also checked for side impacts [13].

The PIPER HBM and the PIPER tool are continuously evolving since its release (v1.0.0). Compared with the release version, the head model used in the current study has been updated. The tentorium geometry now is updated to be more anatomically accurate; the porous skull bone is meshed with two layer hexahedral elements instead of one layer; the dura mater and pia mater incorporate nonlinear and viscoelastic properties. The material properties of the updated head model are provided in S4 File, while material properties of the neck and all other body components are the same as in the release version of the PIPER HBM.

**Fig 4:**
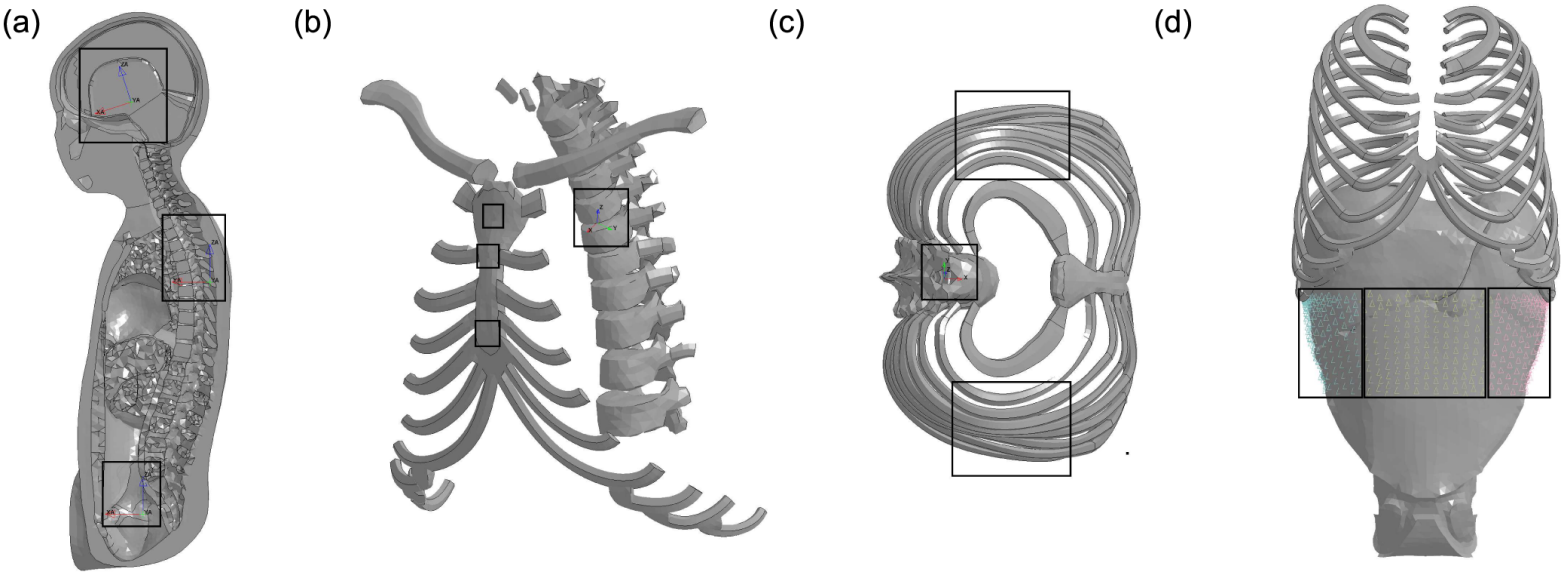
Virtual sensors implemented in the PIPER scalable HBM. (a) Location of the accelerometers to measure head, chest and pelvis acceleration; (b) constrained interpolation of nodes referred to the T9 coordinate system for measuring chest displacement in the sagittal plane; (c) constrained interpolation of nodes referred to the T9 coordinate system for measuring chest displacement in the lateral plane; (d) node sets to measure right, frontal and left abdominal force.

Furthermore, the HBM includes virtual sensors for mimicking the equipped instrumentation in Q-dummies and allows comparisons of accelerations, deflections and forces. Sensors include (1) three accelerometers to measure head, chest and pelvis accelerations (Fig 4a); (2) load cells at each intervertebral disk to measure force and momentum along the spine; (3) constrained interpolation of nodes referred to a coordinate system located to the 9th Thoracic Vertebra (T9) for measuring chest displacement (Fig 4b and 4c); (4) node sets to measure frontal, left and right abdominal force (Fig 4d). Note the head and pelvis virtual sensors have been updated in this study, together with the updated head model are available at the PIPER project website.

### Loading conditions

**Fig 5:**
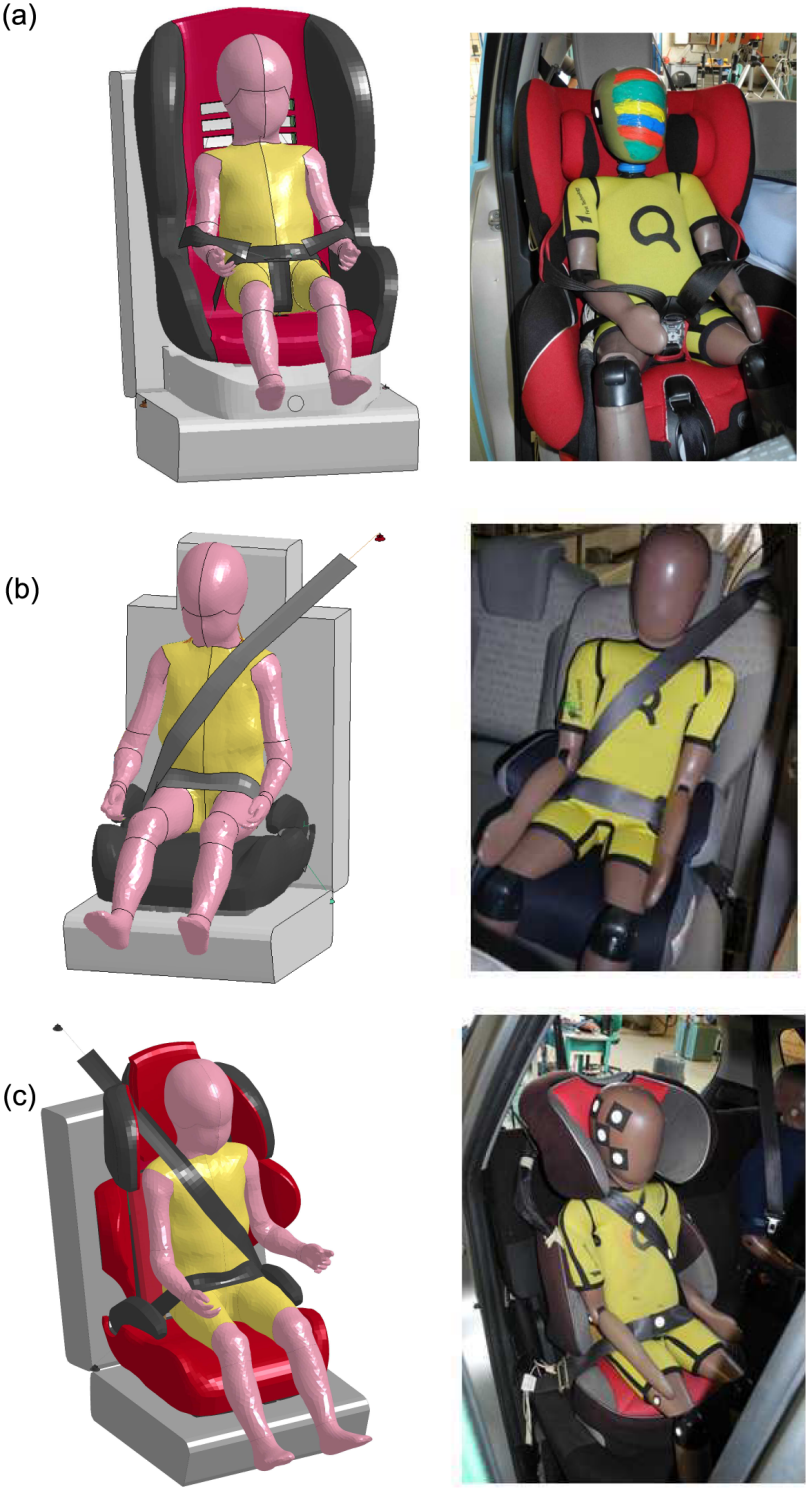
Comparison between the scaled and positioned HBM in the simplified environment and the respective Q-dummy in the physical accident reconstruction. (a) Case 2012; (b) Case 2017; (c) Case 2043.

Simulations were performed with the HBM seated on a CRS model, which in turn was positioned in the PIPER generalized car environment. As shown in Fig 5, the child was restrained by a five-point belt for Case 2012 (Fig 5a) and by a three-point belt for Case 2017 (Fig 5b) and Case 2043 (Fig 5c). For Case 2012, observations at the scene of the accident showed sliding scratches at the shoulder belt of the CRS, leading to the conclusion that the child had slipped from the shoulder belt before the accident occurred. Thus, the simulation was performed with the shoulder belt initially positioned at the elbow (as in the physical accident reconstruction). The belt model was generated using the belt-fit function of LS-Prepost 4.3 and it included a shell section (elastic material, 50 GPa, thickness 1.2 mm) attached by 1D seatbelt elements. The belt loaded at approximately 9.8 kN per change in length. The initial positions of the belt were similar for both HBMs and Q-dummies (Fig 5). *CONTACT_AUTOMATIC_SURFACE_TO_SURFACE card was used in LS-Dyna to simulate the contact between the child and the environment (CRS, seat, seatbelt). For the contact between the child and the seatbelt a dynamic friction coefficient of 0.1 was chosen. The static friction coefficient was 0.1. Also, the 2D seatbelt elements were fully integrated to avoid hourglass distortions.

The FE models were loaded by applying X, Y and Z pillar accelerations to the rigid components of the car environment. For reproducibility, input data can be found in supporting files S1, S2 and S3 Data.

### Age scaling and positioning

In the PIPER tool, scaling methodologies are based on Kriging nonlinear interpolation [17]. Target of the scaling are main anthropometric dimensions based on GEBOD regressions, which are represented by a network of carefully selected control points. The control points drive the model morphing and regressions can be calculated between 1.5 and 6 y.o. Local changes are represented as additional constraints: for the neck, a set of internal control points drive the change of local features such as the angle of the facet joints, the dimensions of the body of the vertebras and the length of the cervical spine. Similarly, anatomical landmarks, such as the glabella, opistocranion, tragus, vertex, nasion and mental protuberance, drive the transformation for the head in order to represent variations in segment proportions with age. In the current study, child scaling was performed with the PIPER tool (v1.0.1) to scale the baseline model (6 y.o.) to a 26 m.o. child for Case 2012 and to a 5 y.o. child for Case 2017 and Case 2043. The child module was used targeting the specific age of the child involved in the accident (Fig 1c).

**Fig 6:**
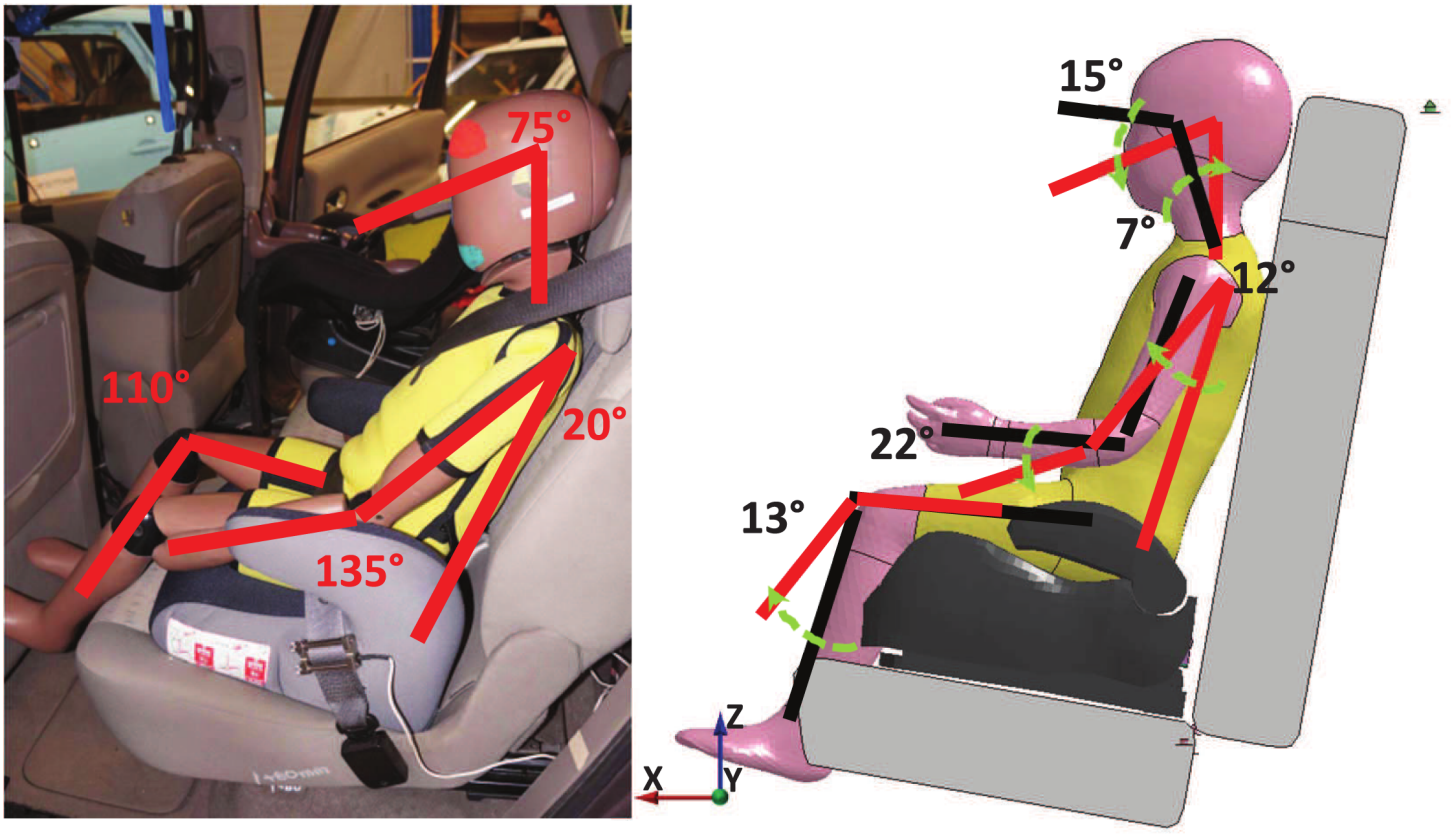
Example of how kinematics chains for positioning were estimated from available documentation of Case 2017. Similar procedures were used for Case 2012 and Case 2043. Rotations of body parts along the Y direction were sufficient to position the HBM close to the Q-dummy.

In the PIPER tool, positioning methodologies are based on lightweight physics simulation approaches. They allow simulating the dynamics of interacting objects using abstract equation solvers in real-time. The HBM is imported in the PIPER tool via metadata and divided in deformable (flesh) and rigid (bone) components. Also, bone collisions can be activated to avoid penetration of rigid components during the motion. The pre-position can then be used to update the model mesh or exported to serve as a basis for a full positioning simulation. In the current study, the pre-positioning module of the PIPER tool was used for positioning of the HBMs and body parts were moved either by relative frame with respect to the global frame or using model joints (Fig 1b). To compare the performances of the Q-dummies to the HBMs, the HBMs were positioned as close as possible to the respective dummy. Kinematics chains were estimated from available data (Fig 6). Table 3 reports the information necessary to reproduce the positioning for each accident reconstruction.

**Table 3:** Angle values used for positioning of the PIPER scalable HBM. Rotations of frames were performed relatively to the y-axis of the world frame. Also, joint rotation was performed along the y-axis of the joint frame.

Frame Name (Relative to World Frame)	Angle Value (ry)		
	Case 2012	Case 2017	Case 2043
Atlas	0°	-7°	-7°
Axis	0°	-7°	-7°
Third Cervical Vertebrae	0°	-7°	-7°
Fourth Cervical Vertebrae	0°	-7°	-7°
Fifth Cervical Vertebrae	0°	-7°	-7°
Sixth Cervical Vertebrae	0°	-7°	-7°
Seventh Cervical Vertebrae	0°	-7°	-7°
Skull	15°	15°	15°
**Joint Name**			
Left Hip	15°	0°	0°
Right Hip	15°	0°	0°
Left Glenohumeral	0°	-12°	10°
Left Elbow	27°	22°	-11°
Left Wrist	0°	0°	0°
Right Glenohumeral	0°	-12°	-10°
Right Elbow	27°	22°	5°
Right Wrist	0°	0°	0°
Left Knee	-18°	-13°	-17°
Left Ankle	0°	0°	0°
Right Knee	-18°	-13°	-17°
Right Ankle	0°	0°	0°

## Results

**Fig 7:**
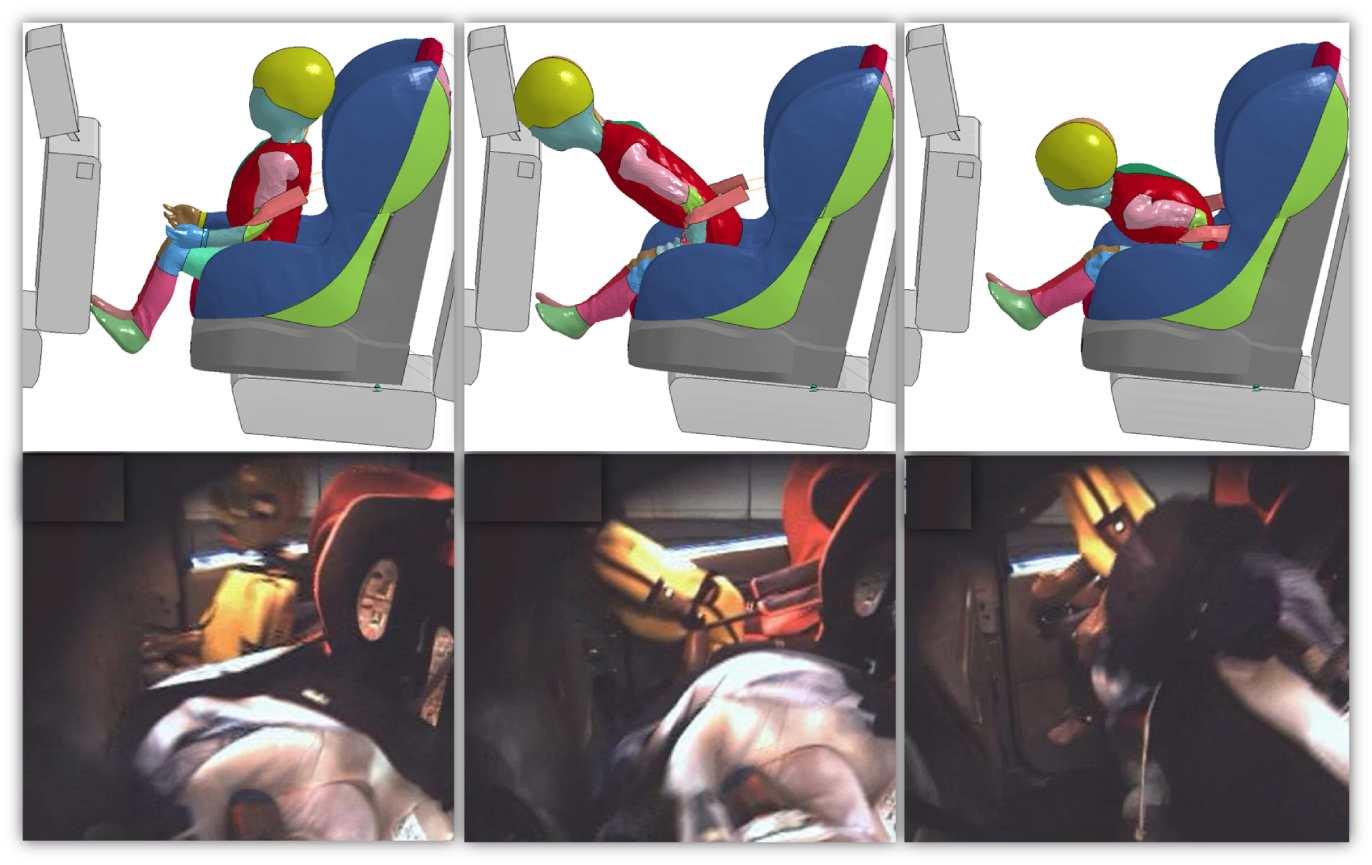
Comparison of the kinematics of the HBM during computer simulation (top) and the dummy during physical accident reconstruction (bottom) for Case 2012. The images in the top row were captured at 71 ms (left), 104 ms (middle) and 137 ms (right) respectively and the HBM kinetics during the simulated entire impact is provided in S1 Movie.

**Fig 8:**
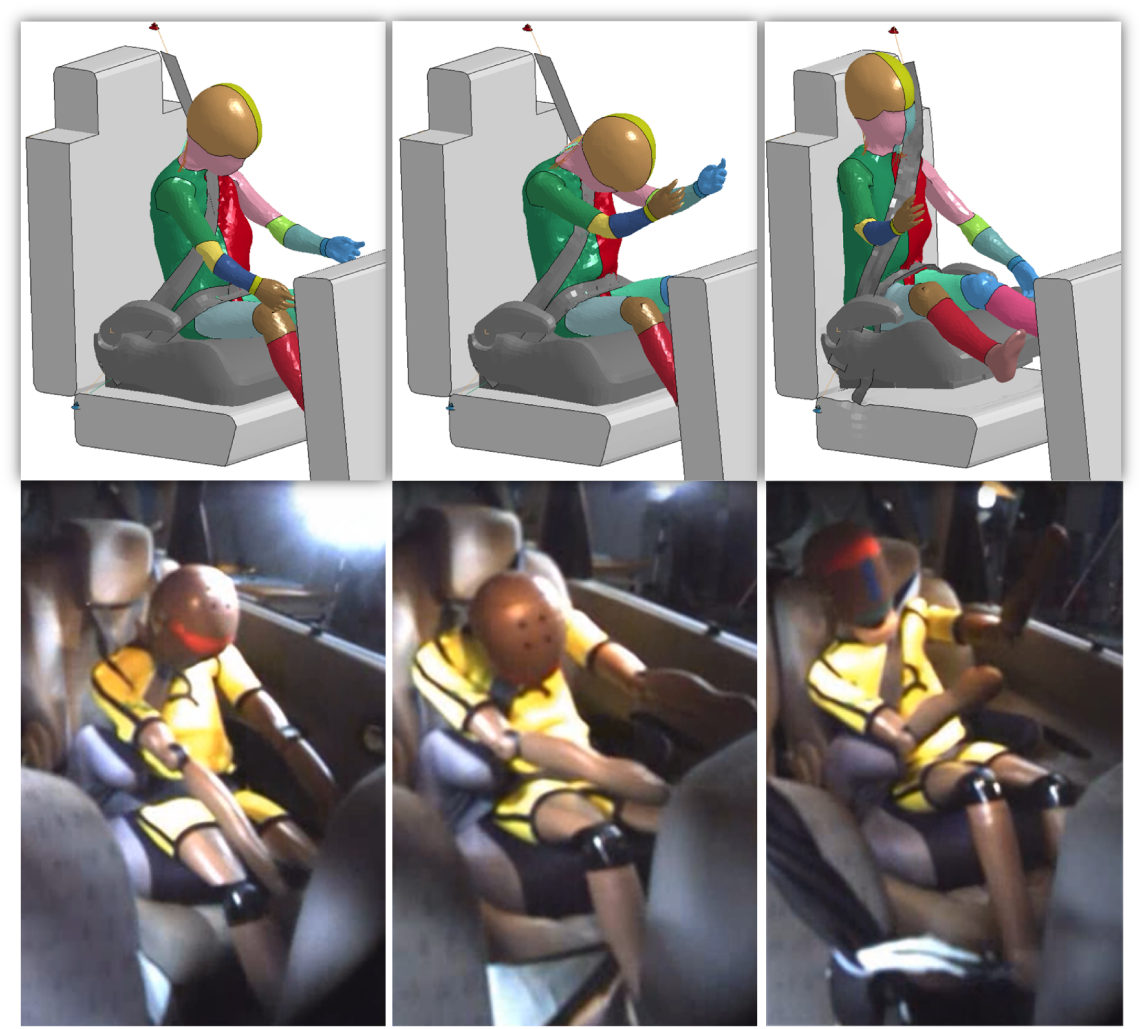
Comparison of the kinematics of the HBM during computer simulation (top) and the dummy during physical accident reconstruction (bottom) for Case 2017. The images in the top row were captured at 70 ms (left), 96 ms (middle) and 217 ms (right) respectively and the HBM kinetics during the simulated entire impact is provided in S2 Movie.

**Fig 9:**
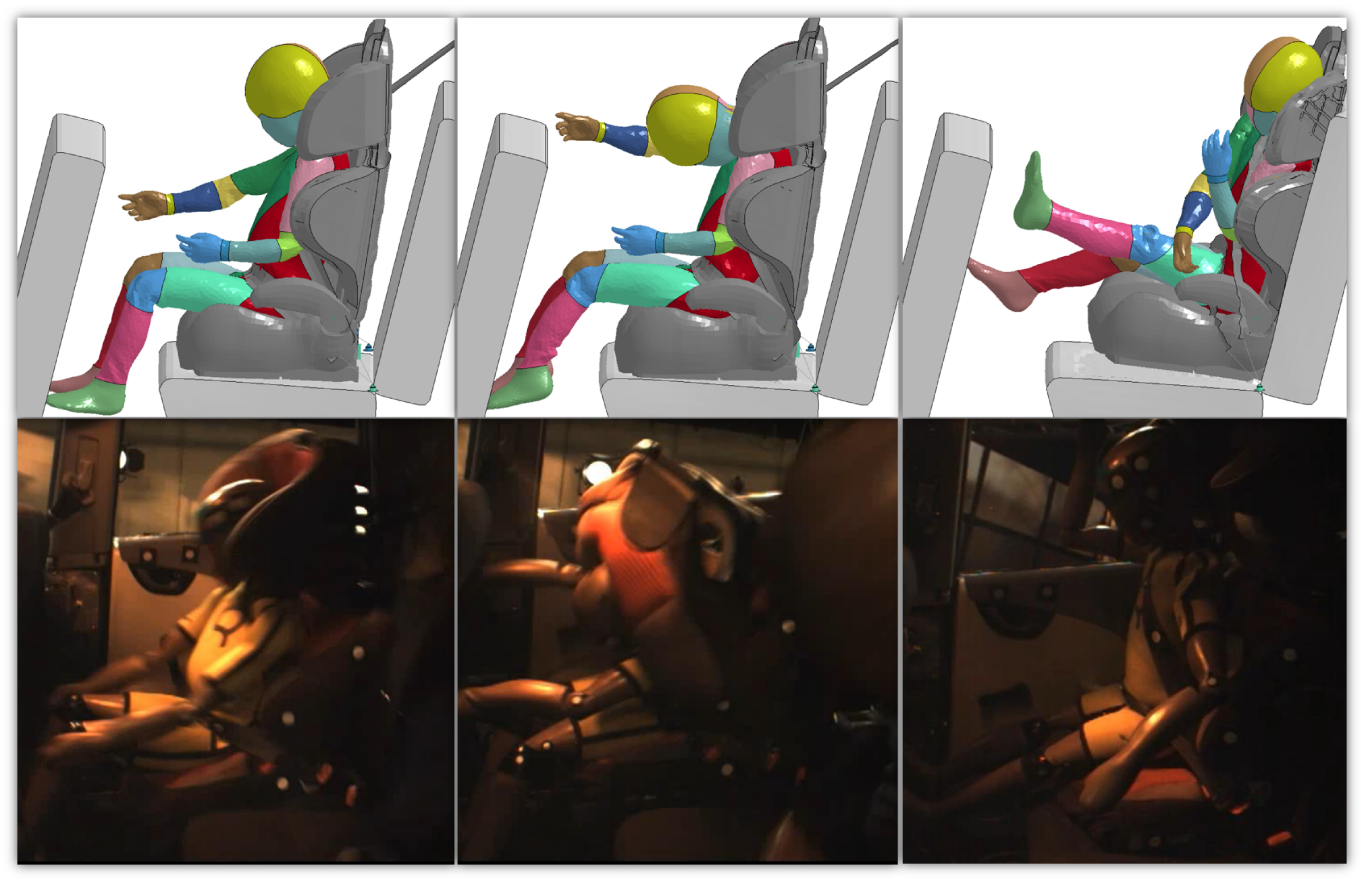
Comparison of the kinematics of the HBM during computer simulation (top) and the dummy during physical accident reconstruction (bottom) for Case 2043. The images in the top row were captured at 60 ms (left), 81 ms (middle) and 260 ms (right) respectively and the HBM kinetics during the simulated entire impact is provided in S3 Movie.

Figs 7–9 represent the comparison of the kinematics of the HBM during computer simulation and the dummy during physical accident reconstruction. The figures show the braking, crash and rebound phase of the accident. It can be noticed that the dynamic of the events followed similar trend between the Q-dummies and the HBMs. In Case 2012 (Fig 7), at first, the upper part of the body extended. Secondly, the head impacted the front seat and, finally, the child bounced back constrained by the seatbelt. Some mismatch between the HBM and the dummy can be seen especially in the last phase, where the HBM seemed to bend more forward and was compressed to a greater extent than the dummy. In Case 2017 (Fig 8), similarly, the upper and lower extremities extended first, then the body hanged on the seatbelt while the head bended forward. Finally, the child bounced back hitting the rear seat. A minor mismatch between the HBM and the dummy can be noticed during the rebound of the body, where the HBM assumed a more neutral position while the dummy bended towards the right side of the car. In Case 2043 (Fig 9), the body sustained strenuous load. At first, the upper extremities extended. Secondly, the trunk bended forward followed by the CRS. Finally, the child flew violently hitting the backrest of the seat with visible motion along the vertical axis as well as extension of upper and lower extremities. Some mismatch can be observed during the crash phase. The seatbelt in the physical accident reconstruction seemed to be looser than in the HBM simulation, allowing the CRS and the child to move a longer distance in the X-axis. Also, the upper body extrusion in the Q-dummy was larger than the HBM.

**Fig 10:**
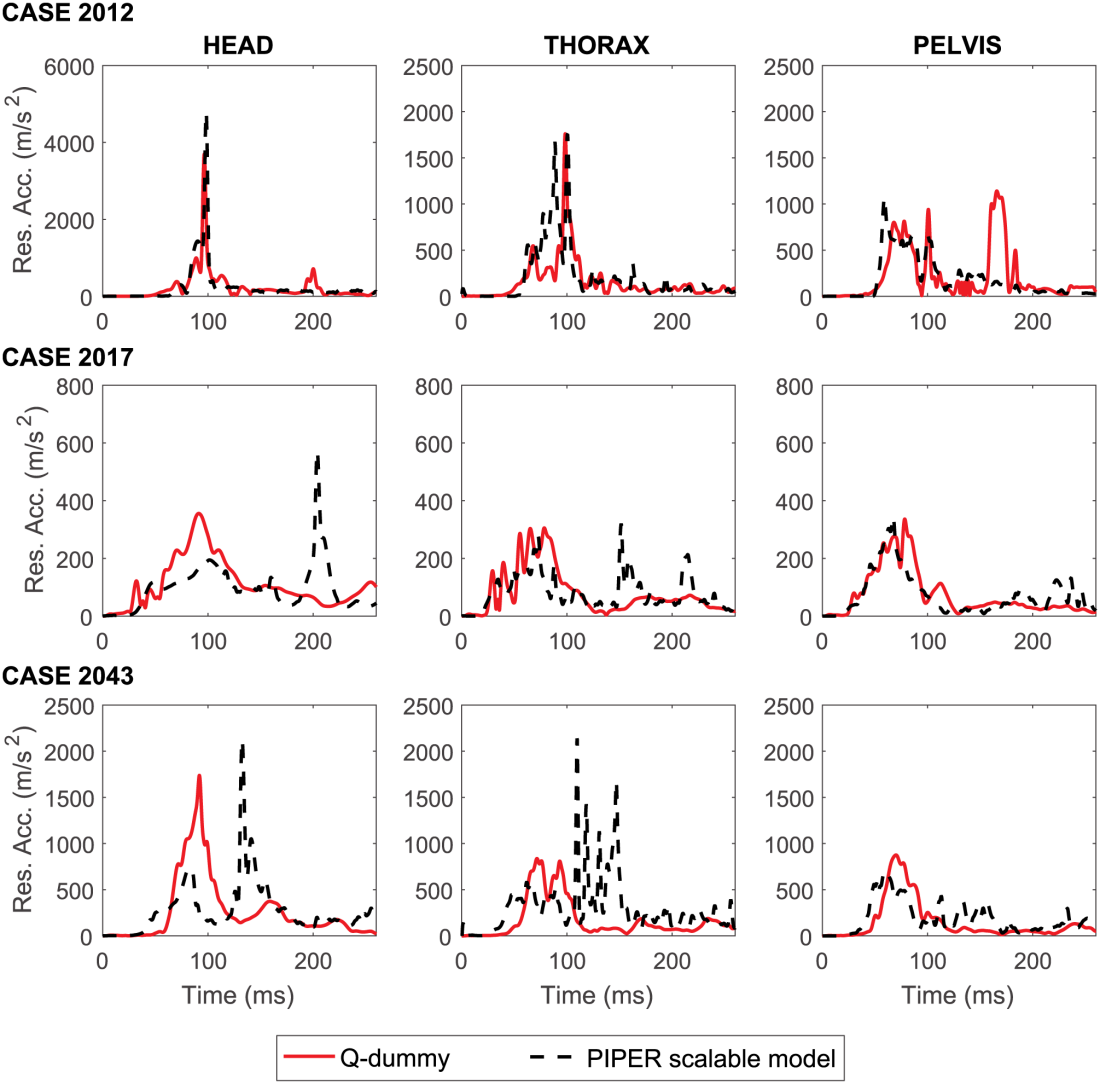
Comparison between the resultant accelerations of head, thorax and chest in the HBMs (dashed black lines) and dummies (full red lines) for all the performed accident reconstructions. Both the HBM and Q-dummy curves were processed by a low-pass Butterworth filter using the same cutoff frequency of 180 Hz.

Fig 10 reports the comparison between the resultant accelerations of head, thorax and pelvis in the HBMs and dummies for the three reconstructed accidents. In Case 2012, the head of the child impacted severely the front seat due to the slipped CRS belt (see S1 Movie) resulting in a large acceleration in the head, both in the HBM and the dummy. The thorax and pelvis accelerations predicted from the HBM were comparable with the dummy readings. In case 2017 and 2043, the HBM simulations showed two major peaks of acceleration (Fig 10); the first acceleration peak occurred during the crash phase, and the second occurred during the rebound phase. For thorax and pelvis, the first peak was comparable with the Q-dummy both in phase and magnitude, but the peak in the head was lower in the HBM simulation than in the dummy. Both cases 2017 and 2043 had a large second acceleration peak in the head and thorax from the HBM simulation: this was due to the rebound of the head and the upper body hitting the backrest of the car seat (see S2 and S3 Movies).

**Fig 11:**
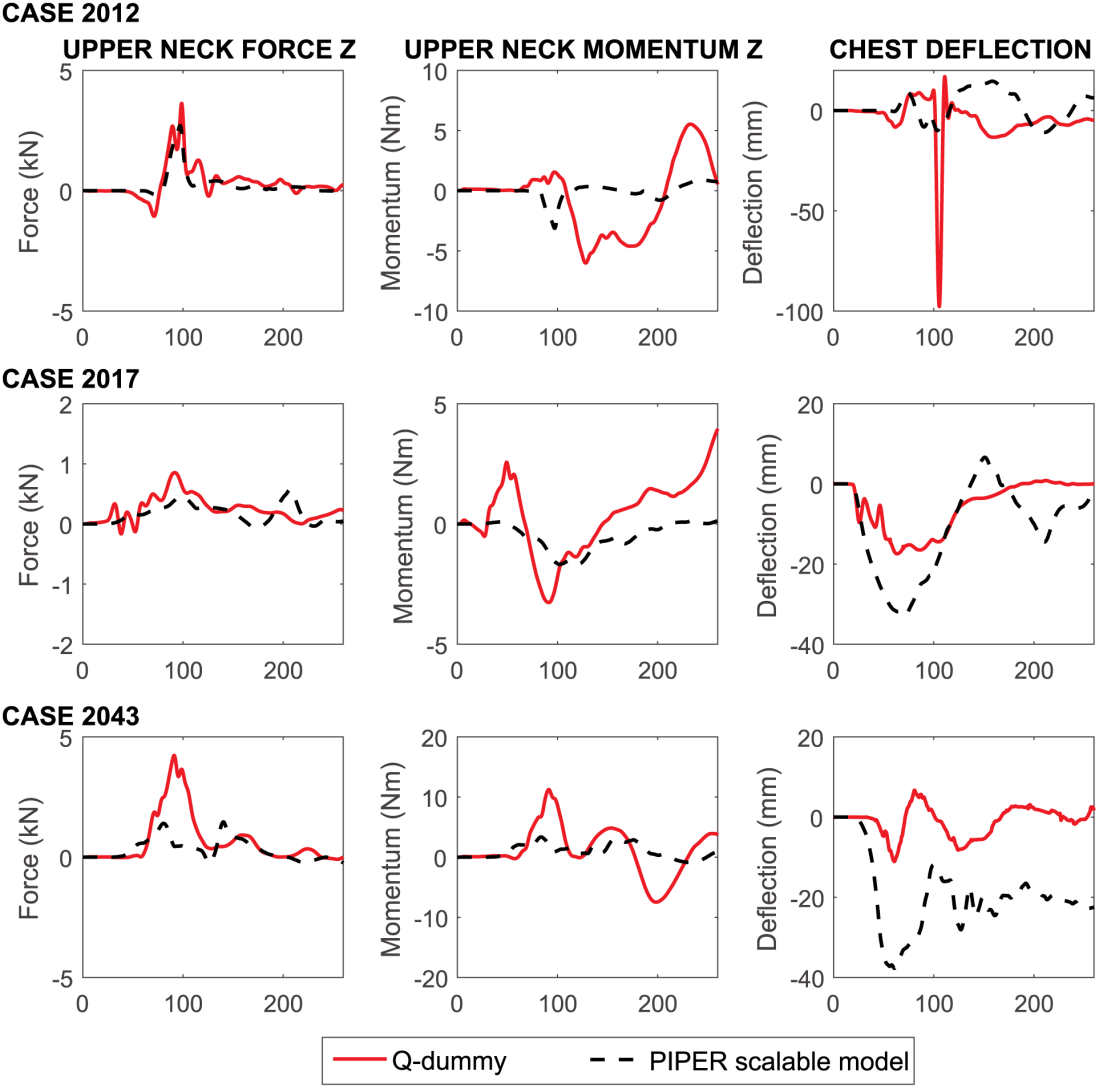
Comparison between the upper neck force, upper neck momentum and chest deflection in the HBMs (dashed black lines) and dummies (full red lines) for all the performed accident reconstructions. Both the HBM and Q-dummy curves were processed by a low-pass Butterworth filter using the same cutoff frequency of 180 Hz. The large chest displacement measured in the Q-dummy in Case 2012 appeared to be some noise.

Fig 11 represents the comparison between the upper neck force, upper neck moment and the chest deflection in the HBMs and dummies. Forces transmitted across the horizontal plane between the 2nd Cervical Vertebra (C2) and C3 were generally comparable. However, the load cell of the dummy showed typically larger momentum than the HBM. In contrast, sagittal plane chest displacement (chest deflection) was larger in the HBM than the dummy, especially peak differences up to 2.6 centimeters (cm) were seen in Case 2043. More comprehensive comparisons between the HBMs and Q-dummies, including acceleration components in X, Y and Z directions, CRS accelerations, belt forces, neck force, neck momentum components, and abdomen pressure are provided in S5 File.

**Fig 12:**
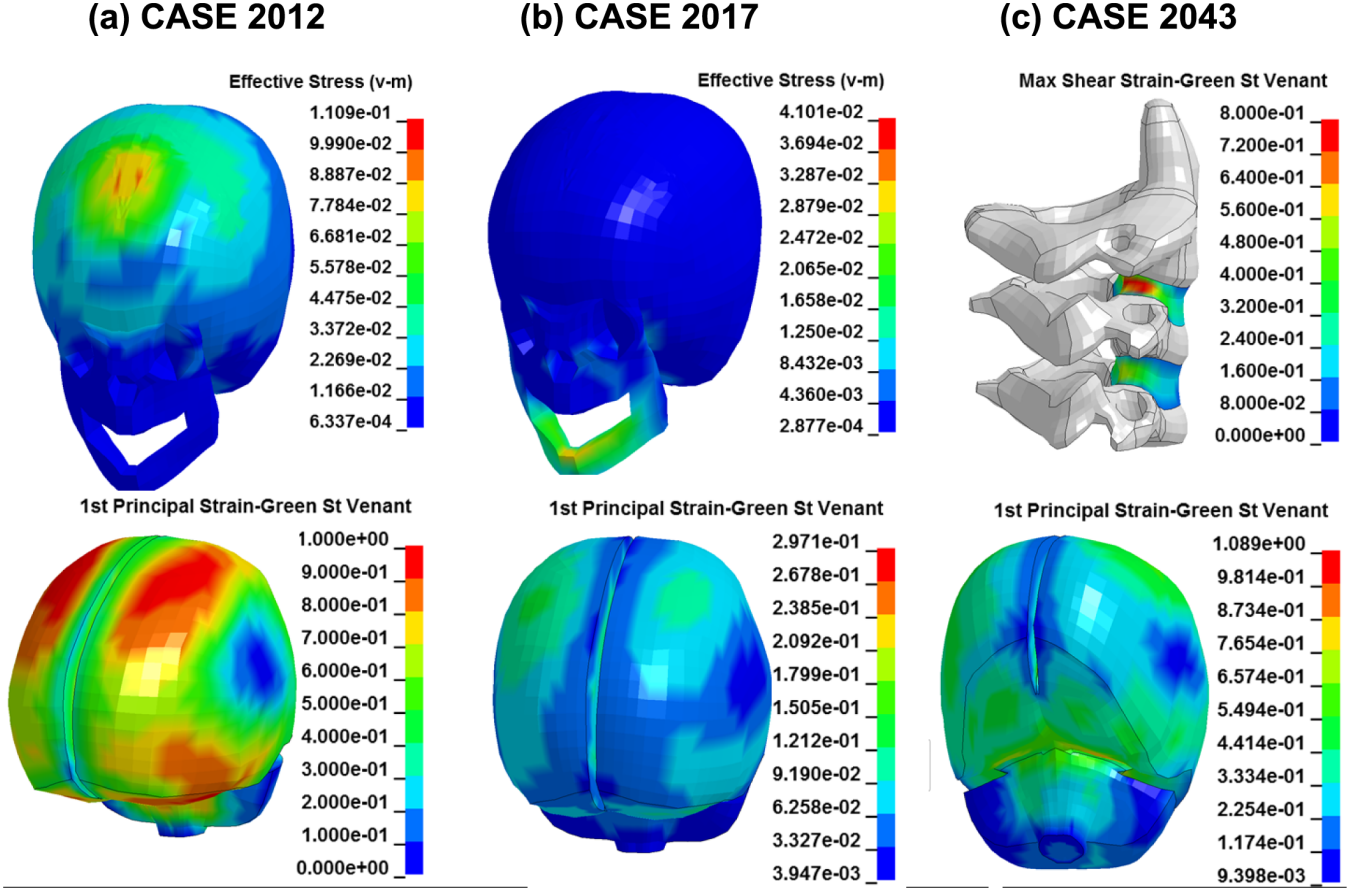
Maps of von Mises stress in the skull (unit is GPa in the legend), first principal strain in the brain, and maximum shear strain in the cervical disk that sustained injury in the real accident.

Several computational studies have shown correlation between von Mises stress/Principal Green-Lagrange strain and traumatic injury [18–22]. In particular, von Mises stress has been associated to bone fracture while Principal Green-Lagrange strain can be a good indicator of brain or spinal injury. To analyze the capability of the PIPER scalable HBM in injury prediction, maps of von Mises stress, first principal and maximum shear Green-St Venant strain were extracted for the areas of the body that sustained injury in the real accident. According to the accident documentation, in Case 2012, the child sustained face and scalp hematomas, a fracture of the left orbit, extra-dural frontal hematoma, contusion of the frontal lobe and hemorrhage in the meninges. Fig 12a depicts the HBM stress and strain predictions in the head when values reached maximum. Areas of large stress were located in the frontal lobe, close to the injured body parts. In particular, the maximum von Mises stress reached a value of 110.9 MPa, which was compatible with skull fracture level according to [18–20]. The predicted injury location was the area of the frontal bone just above the eye orbits. The maximum principal strain reached over 100% in the frontal lobe of the brain close to the skull fracture, indicating major brain injury associable to death [21, 22]. In the accident, in contrast, the child recovered well.

Fig 12b represents the skull stress and brain strain predictions for Case 2017. According to the accident documentation, the child did not report any injury. In the simulation, the maximum von Mises stress reached a value of 41.0 MPa at the level of the chin when the head impacted the upper body during the rebound phase (see S2 Movie). The maximum principal strain values reached 29.7%, indicating a risk of concussion based on previous accident reconstructions using the same constitutive properties for the brain in adults [21].

Fig 12c shows the stress and strain maps for Case 2043. According to the accident documentation, the child had major injuries all over the body and did not survive. Injuries included diffuse axonal head trauma, dislocation of the cervical spine (C3), thorax trauma, laceration of several internal organs and fracture of the clavicula. For the brain, the maximum principal strain was located in the temporal lobe at the level of the skull base and it reached values of 108.9%. This indicated extensive brain injury and was compatible with the MAS 6 injury and the death of the child [21, 22]. For the dislocation of the C3, the maximum shear strain in the nucleus pulposus and ground substances neighboring to the C3 showed values of above 80%. Also the peak z-force transmitted across the disk between the C2 and the C3 was around 1500 N. According to [23], a force of 720 N is sufficient to cause failure in the vertebrae of the cervical spine (5 y.o.). Thus, the model response was in agreement with bone failure [23]. Finally, injuries to the thorax were compatible with the peak acceleration predicted by the model (Fig 10), according to [24] and [25] a load of around 117 g could cause extensive thorax trauma.

## Discussion

Three accident reconstructions were simulated to investigate the dynamic performances of the PIPER scalable HBM. Scaling and positioning of the model were performed using the PIPER tool to account for different ages and positions of the child in the accidents, all starting from the same mesh connectivity [13]. The scaling and positioning were completely interactive and user-friendly, and as shown in previous studies [13], the quality of the derived HBMs was close to the quality of the baseline model. This study highlights the validity of the scalable/posable approach for real accident reconstructions and attests the feasibility to use the PIPER scalable HBM for determining child injury injury tolerances/risk curves based on accident reconstruction.

Human injury tolerances/risk curves indicate the occurrence of injury as a function of a biomechanical metrics in response to external mechanical loads and are often developed based on various statistical models [26]. The approach developed in [3] for Q-dummies could be successfully expanded to the use of HBMs. Unlike dummies, which are only available in few dimensions and ages, the PIPER scalable HBM can be scaled to the same dimensions as the occupant in the accident. Further, the HBMs allow accurately representing the complex anatomy, material properties of the human body and the growth with age. Therefore, injury risk curves derived using HBMs, due to the higher biofidelity, can better predict injuries compared with using Q-dummies. However, to derive risk injury curves, results from more numerical accident reconstructions are necessary, also more investigation on issues of model performances, uncertainty and sensitivity the accident reconstructions is needed. This study represents a first attempt to determine child injury tolerances based on the PIPER scalable HBM.

The comparison between Q-dummies and HBMs showed similarities and differences. Although the dynamic of the accidents was reconstructed in a similar way independent of the used model (Figs 7–9), differences were visible in terms of peak accelerations and forces measured along the spine. Typically, HBMs showed higher flexibility and compressibility than dummies. Especially, differences in flexibility was seen at the level of the cervical spine, head and chest. Also larger differences were observed after the interaction of the model with the front seat or the seatbelt, indicating possible discrepancies between the responses of the dummies and HBMs. In particular, chest deflection differed importantly (2.6 cm). This observation was in agreement with a previous study [6], where it was highlighted that the deflection sensor of the Q-dummy largely underestimated the chest deflection when belt loading was applied to the upper ribcage. It was suggested that this is due to the location of the sensor, which is implanted in an unrealistically stiff region of the dummy. The use of an HBM can therefore facilitate the measure of deformation, improving the prediction of injury.

It should be noted it was not the intention to perfectly reproduce dummy readings using HBMs, due to the inherent differences between the models. However, dummy readings provided a valuable reference to check the numerical reconstructions using HBMs, especially where similar performances should be expected. For example, the pelvis acceleration predicted from the HBM was close to the respective dummy (Fig 10), indicating the interaction between the CRS and the car was modeled properly. This in turn ensured that the loading to the child HBM reflected what happened during the real-life crash, since the installation of CRS during the physical reconstructions was based on in-depth investigations of the real accident by experts.

To further analyze the capability of the PIPER scalable HBM in injury prediction, maps of von Mises stress, first principal and maximum shear Green-St Venant strain were extracted for the areas of the body that sustained injury in the real accident. In the past decades, several computational studies have shown correlation between stress/strain and traumatic injury [18–22]. Mechanical predictors of bone fracture are typically maximum von Mises stress, maximum shear stress and maximal first principal stress. Mechanical predictors of brain or spinal trauma are instead Principal Green-Lagrange strain or shear strain. In this study we focused on the local mechanical behaviors of the head and neck during the accident, and compared HBM predictions of skull/vertebra fracture and brain/spinal injury to actual injuries from the real-life accidents. Overall, the PIPER scalable HBM managed reasonably well to predict the injury severity and location for the real-life accidents. However, according to the human tolerances currently available in the literature, principal strain values in the brain were found to over-predict the risk of injury in Case 2012. This phenomenon was already observed in another study [27] where simulation with adapted child HBMs showed higher brain tissue strain responses than measured in adults. In this study, possible reasons for these large strain predictions could be associated to the peculiar dynamic of the accidents: for Case 2012, for example, an extremely violent impact occurred to the head, with the child hitting a metal bar under the foam covering of the front seat. This impact produced very high energy which numerically translated in large strain in the brain. Also, separation between gray and white matter was not implemented in the brain model and could have produced higher strain values. It is important to remember that human tolerances from the literature refer to adults. Youths may be able to accommodate larger deformation because they are more flexible. An opposite possible conclusion is that youth are at a higher risk of injury than adults. Currently, there is not sufficient biomechanical information to suggest that any of these two claims should prevail and further analysis is necessary before child body tolerances to external loads could be established.

In the current study, the effect of age on material parameters was not considered and the same material properties were used in all aged models. In future studies focusing on age-dependent injury mechanisms, age-dependent material properties could be implemented.

## Conclusion

This study proved the feasibility of combining the PIPER scalable HBM and the PIPER tool for performing child accident reconstruction. The scalable/posable approach was very powerful and enabled to perform computer simulations with HBMs of different ages and in different positions. Overall, the PIPER scalable HBM managed reasonably well to predict the injury severity and location for the reconstructions. However, more investigation is needed to evaluate the dynamic performances of the model. Following this preliminary investigation, injury tolerances could be estimated based on the full CASPER accident reconstruction database. The results also indicate the usability of the PIPER scalable HBM combining with the PIPER tool for evaluating the performance of CRS during the development process.

## Supporting information

S1 File In detail description of accident Case 2012 from the CASPER accident reconstruction database. The pdf file reports details about the accident circumstances, the vehicles and the child occupant analyzed in this paper.
(PDF)

S2 File In detail description of accident Case 2017 from the CASPER accident reconstruction database. The pdf file reports details about the accident circumstances, the vehicles and the child occupant analyzed in this paper.
(PDF)

S3 File In detail description of accident Case 2043 from the CASPER accident reconstruction database. The pdf file reports details about the accident circumstances, the vehicles and the child occupant analyzed in this paper.
(PDF)

S4 File Material properties of the head components. (DOCX)

S5 File Compiled supplementary figures for Cases 2012, 2017 and 2043. (DOCX)

S1 Movie Animations of HBM kinetics during the simulated entire impact for Case 2012. (MP4)

S2 Movie Animations of HBM kinetics during the simulated entire impact for Case 2017. (MP4)

S3 Movie Animations of HBM kinetics during the simulated entire impact for Case 2043. (MP4)

S1 Data Sensor and dummy readings for Case 2012 from the CASPER accident reconstruction database. This zip folder contains input accelerations for reproducibility of the accident reconstruction. Also, the readings from the dummy sensor are reported for reproducibility of the calculations.
(ZIP)

S2 Data Sensor and dummy readings for Case 2017 from the CASPER accident reconstruction database. This zip folder contains input accelerations for reproducibility of the accident reconstruction. Also, the readings from the dummy sensor are reported for reproducibility of the calculations.
(ZIP)

S3 Data Sensor and dummy readings for Case 2043 from the CASPER accident reconstruction database. This zip folder contains input accelerations for reproducibility of the accident reconstruction. Also, the readings from the dummy sensor are reported for reproducibility of the calculations.
(ZIP)
