# Supplementary material for: Performances of the PIPER scalable child human body model in accident reconstruction
Source: PLoS One. 2017 Nov 14;12(11):e0187916. doi: 10.1371/journal.pone.0187916 (PMC5685610; doi:10.1371/journal.pone.0187916)
Supplement: S1 File — The pdf file reports details about the accident circumstances, the vehicles and the child occupant analyzed in this paper. (PDF) [file pone.0187916.s001.pdf]

Casper Case N° 2012

Accident reference n°: 18301 **Origin : LAB CEESAR**

Accident date : November 2009

Events description:

On a wet road, and due to a too high speed, the driver of a BMW loses the control of his car at the end of a curve (left). The car slides into the lane dedicated to the circulation in the opposite direction in which a Renault Megane Scenic is arriving. The Scenic impacted the BMW at the right rear door level.

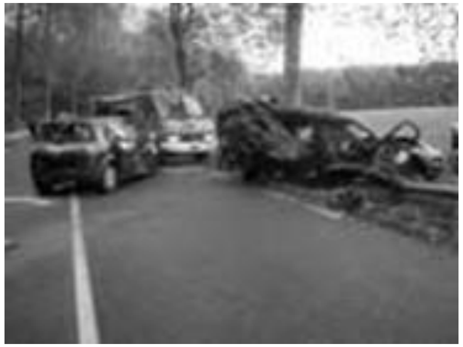

ACCIDENT CIRCUMSTANCES

**Vehicle n°1**	**RENAULT SCENIC II**	**FRONTAL IMPACT**
Year model: 2008 CDC: 12FDEW4	750 mm	EES: 60 km/h
Mass : 1430 kg	Maxi deformation:	DeltaV : n/k km/h

occupants

31 Y M Seatbelt and AB

26 Y F Seatbelt

3 W F Carricot Carrycot

<u>26 M F FWD G1 - harness</u>

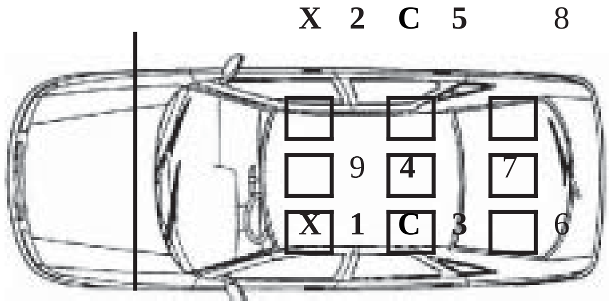

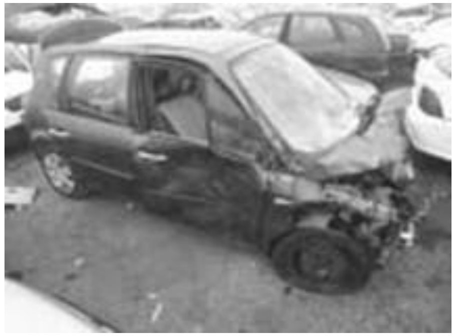

VIEW OF VEHICLE N°1

**Vehicle n°2**	**BMW 525 tds**	**RIGHT SIDE IMPACT**
Year model: 2002 CDC: 04RZEW3	540 mm	EES: n/k km/h
Mass : 1565 kg	Maxi deformation:	DeltaV : n/k km/h

25 Y occupants

M Seatbelt

25 Y F S

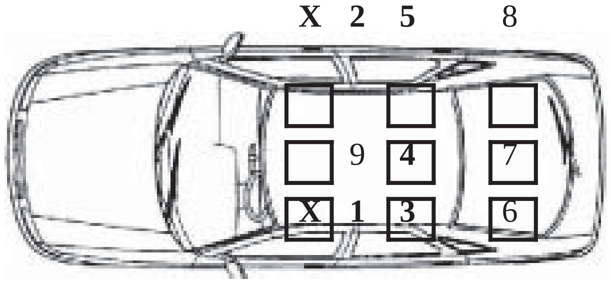

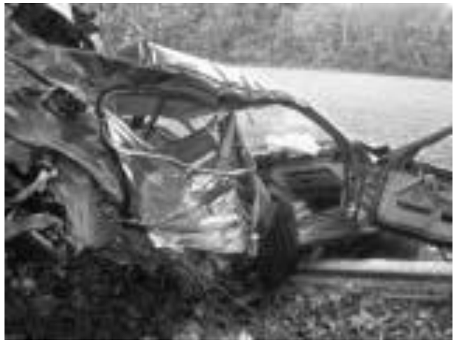

VIEW OF VEHICLE N°2

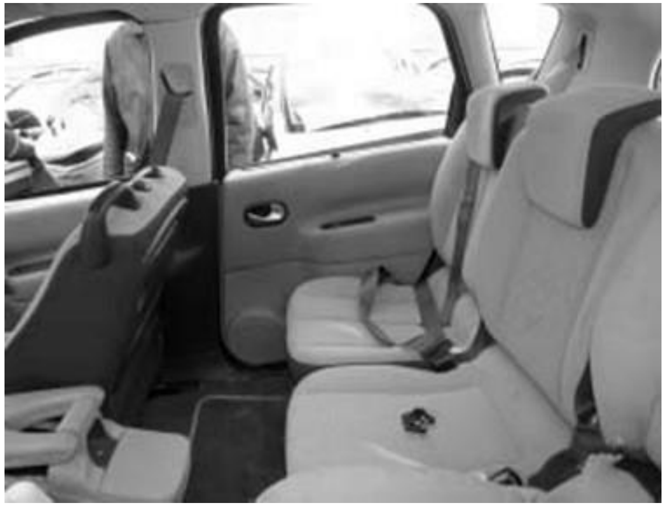

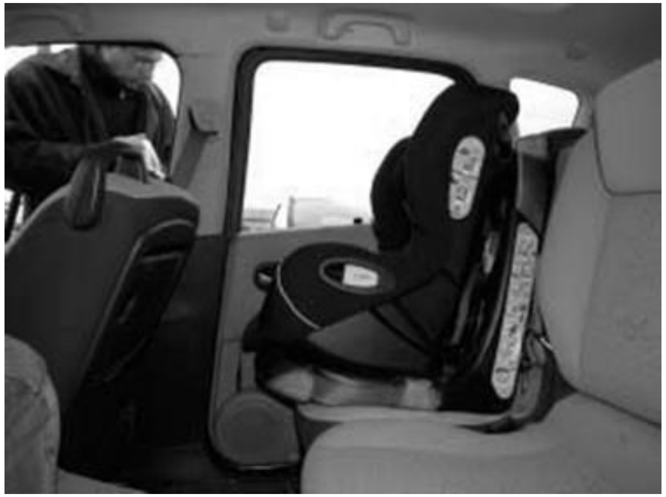

Casper Case N° 2012

SEATING POSITION:		Age: 26 months
**REAR RIGHT**		**88 cm – 14 kg**
Type of restraint:	Use	M.AIS
**FWD FC Harness BEBECONFORT AXYSS (collected)**	**YES**	**4**

Injuries

Injury	Cause	AIS
Left eye peri-orbital contusion	Right front seatback	2 1 04 02.1
Face and scalp hematomas	Right front seatback	1 1 04 02.1
Fracture - roof the orbit (L)	Right front seatback	1 5 02 02.3
Fronto parietal embarrure	Right front seatback	1 5 04 08.4
Pneumencephalia		1 4 06 82.3
Extra dural – frontal hematoma (L)	Right front seatback	1 4 06 30.4
Hemorrhagic contusion of the frontal lobe – left side	Right front seatback	1 4 06 29.4
Meningia heamorraghe	Right front seatback	1 4 06 84.3
R Humerus displaced fracture (metaphyso-diaphysar) with contusion of the radial nerve	Arm stuck in harness?	7 5 26 06.3

CRS has been anayzed (with CRS manufacturer) and collected.
The fixation of CRS to the car is correct.
The girl was wearing a winter coat (mountain style : sick, soft and slippery). In addition the harness tension was not sufficient to ensure a good maintain of the upper part of the body.
Both harness straps were positioned on shoulders (friction marks on both), but shoulders escaped from harness straps during the crash. As a result, the girl sustained a hard contact of her head with the front seatback. In the area of the impact, when removing the foam a steel tube of the seat structure is present and has been the source of the main injuries to the head, face and brain.
The girl well recovered of the accident.
