# Supplementary material for: Performances of the PIPER scalable child human body model in accident reconstruction
Source: PLoS One. 2017 Nov 14;12(11):e0187916. doi: 10.1371/journal.pone.0187916 (PMC5685610; doi:10.1371/journal.pone.0187916)

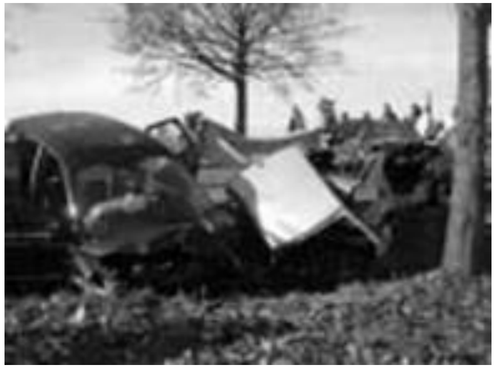

ACCIDENT CIRCUMSTANCES

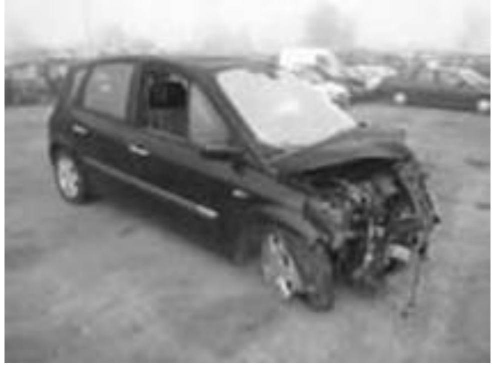

VIEW OF VEHICLE N°1

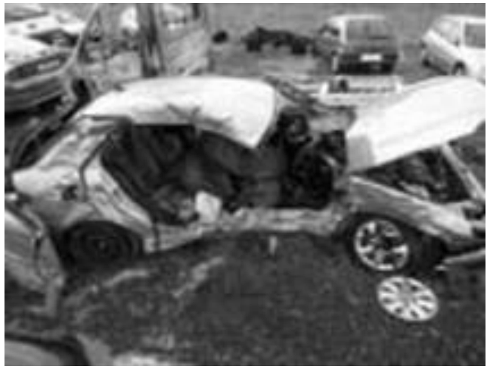

VIEW OF VEHICLE N°2

Casper Case N° 2017

Accident reference n°: 16773 Origin : LAB CEESAR

Accident date :

Events description:

4 vehicles are involved in this accident. The present case is a single collision between vehicle C and vehicle D. Arriving at an intersection, the driver of a P308 (E) suddenly brakes very hard to turn left. A truck (D) following that car cannot stop and collides in its rear, pushing it in the line dedicated for the opposite direction in which a C Xsara (B) was arriving. A sort a side swipe occurs between A and C resulting in a loss of control of C who slides and is stopped in a side impact with a Renault Megane Scenic (A) that had time to break and stop.

Vehicle n°1 RENAULT MEGANE SCENIC FRONTAL IMPACT

Year model: 2003 CDC: 12FDEW3 mm EES: 42 km/h

Mass : kg Maxi deformation: 480 DeltaV : km/h

occupants

34 Y F Seatbelt

5 Y M Booster cushion

5 m M Fwd fc harness

include picture vehicle 1

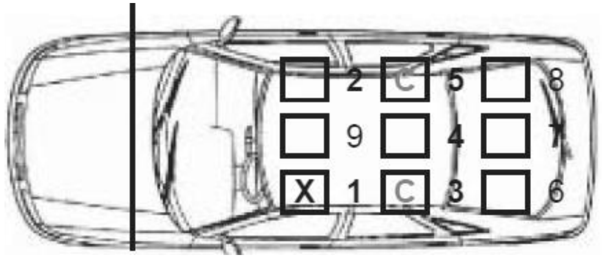

Vehicle n°2 CITROEN XSARA RIGHT SIDE IMPACT

Year model: 2001 CDC: 03REW4 680 mm EES: 42 km/h

Mass : kg Maxi deformation: DeltaV : km/h

occupants

77 Y M Seatbelt

75 Y F Seatbelt

include picture vehicle 2

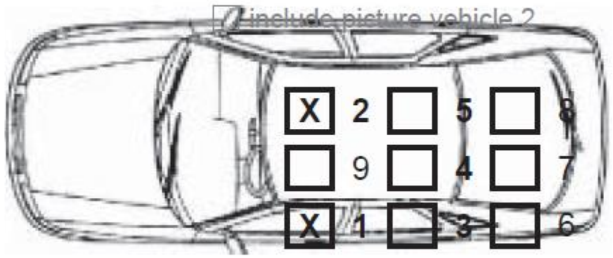

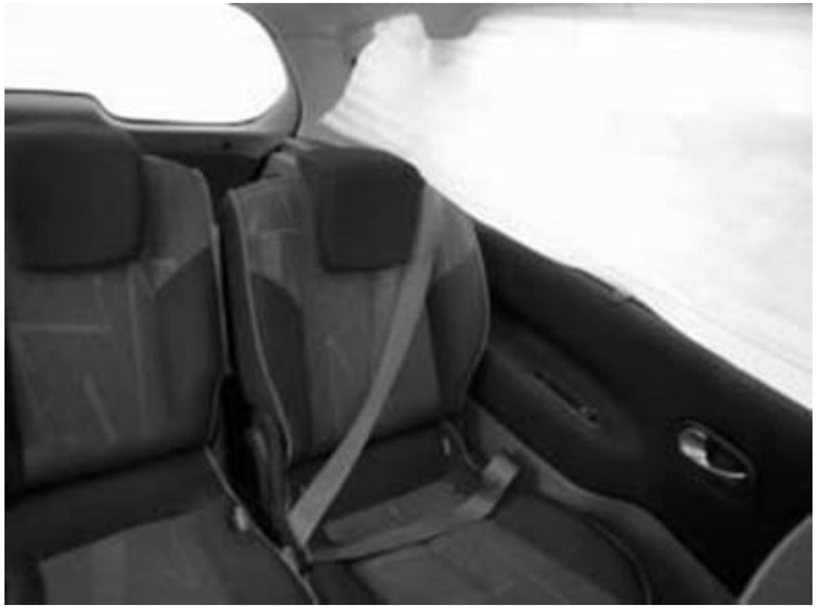

SEATING POSITION:		Age:
**REAR LEFT**		**5 years**
Type of restraint:	Use restraint:	M.AIS
**Booster seat<br>TEAM TEX<br>POLO/UNO<br>E2 03 6018**	**YES**	**0**
		Injuries
		**NOT INJURED**

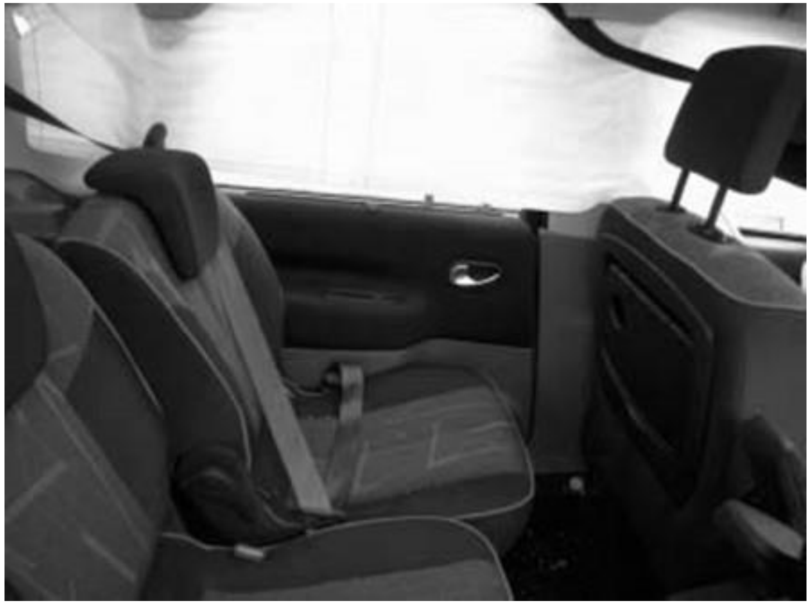

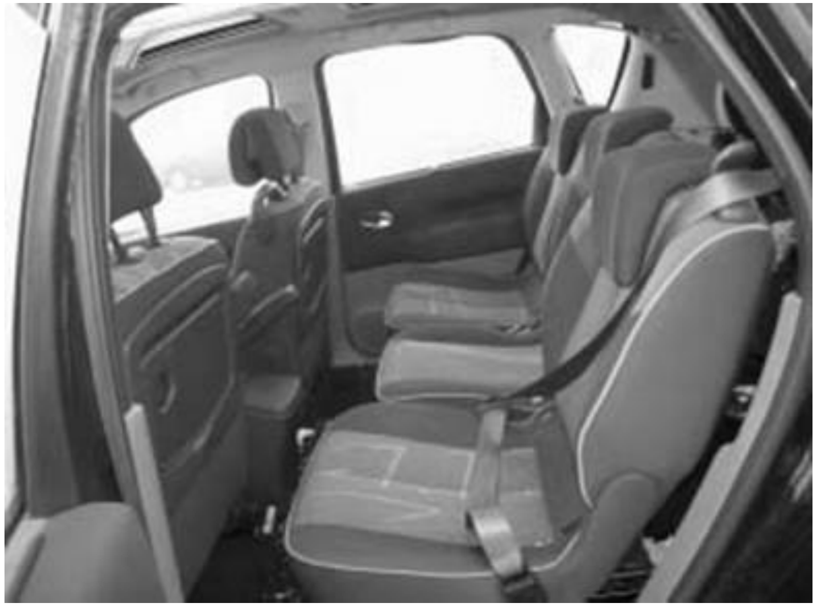

Supplement: S2 File — The pdf file reports details about the accident circumstances, the vehicles and the child occupant analyzed in this paper. (PDF) [file pone.0187916.s002.pdf]
