# Supplementary material for: Performances of the PIPER scalable child human body model in accident reconstruction
Source: PLoS One. 2017 Nov 14;12(11):e0187916. doi: 10.1371/journal.pone.0187916 (PMC5685610; doi:10.1371/journal.pone.0187916)
Supplement: S3 File — The pdf file reports details about the accident circumstances, the vehicles and the child occupant analyzed in this paper. (PDF) [file pone.0187916.s003.pdf]

CASPER Case N 2043

Accident description: the driver of the Honda Jazz with 2 passengers (mother and daughter) drives on a straight rural road. For an unknown reason she suddenly leaves the road to the left and collides frontally with a tree. No skid or brake marks on the road were found.

| | | |
|---|---|---|
| **Vehicle No. 1** | **Honda Jazz 2009** | **FRONTAL IMPACT** |
| Year of model: 2009 | CDC: 12FC0AW6 | EES: 65 (estimation) |
| Mass: 1200 kg | Max. deformation: 890 mm | Delta-v: 75 (estimation) |
| No. of children: 1 | MAIS vehicle: 6 | |

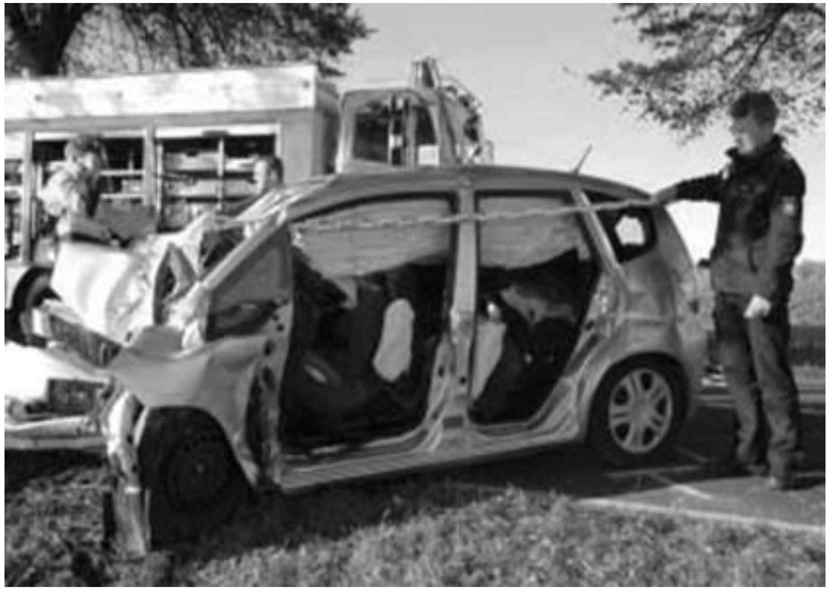

**Occupants**

| | | |
|---|---|---|
| 29Y | F | Seatbelt |
| 49Y | F | Seatbelt |
| **5 Y** | **F** | **FWD facing child seat 15- 36 kg** |

| **Injuries of the 5Y occupant** | | |
|---|---|---|
| **Seating POSITION: Rear right** | **Age: 5 Years** | **MAIS: 6** |
| Type of restraint: FWD facing child seat 15-36 kg<br>**BRITAX RÖMER VIP** | | Use: Yes |

| INJURIES (AIS98) | |
|---|---|
| Diffuse axonal head trauma | 1 4 06 28 . 5 |
| Atlanto-occipital and Atlanto-axial dislocation Cervical spine C3 with Luxation | 6 4 02 34 . 6 |
| Thorax trauma - whole region | 4 1 50 99 . 9 |
| Lung contusion | 4 4 14 10 . 4 |
| Fracture of clavicula | 7 5 22 00 . 2 |
| Laceration of Liver | 5 4 18 20 . 2 |
| Laceration of spleen | 5 4 42 20 . 2 |
| Laceration of left kidney | 5 4 16 20 . 2 |
| Rupture of pancreas | 5 4 29 99 . 2 |
