# Supplementary material for: Performances of the PIPER scalable child human body model in accident reconstruction
Source: PLoS One. 2017 Nov 14;12(11):e0187916. doi: 10.1371/journal.pone.0187916 (PMC5685610; doi:10.1371/journal.pone.0187916)
Supplement: S4 File — (DOCX) [file pone.0187916.s004.docx]

**Supporting Information**

Table A. **Material properties of the head model components in this study. The capital letter E represents the Young’s modulus, and *ν* is Poission’s ratio.**

| Tissue | Material properties | Density (kg/m^3^) |
| --- | --- | --- |
| *Scalp connective tissue* | Ogden 1^st^ order + viscosity  µ_1_=12.98×10^4^ Pa, α_1_=24.23  G_1_=1348 kPa, G_2_=1572 kPa, β_1_=3.03 1/s, β_2_=0.404 1/s | 1133 |
| *Scalp adipose tissue* | Ogden 1^st^ order  µ_1_=3992 Pa, α_1_=8.82 | 1133 |
| *Outer compact bone* | E=9.32 GPa, ν=0.22 | 2000 |
| *Inner compact bone* | E=9.32 GPa, ν=0.22 | 2000 |
| *Porous bone* | E=1.0 GPa, ν=0.24 | 1300 |
| *Brain tissue* | Ogden 2^nd^ order + viscosity  µ_1_=53.8 Pa, α_1_=10.1, µ_2_=-120.4 Pa, α_2_=-12.9 | 1040 |
| *Cerebrospinal fluid* | Bulk modulus K = 2.1 GPa | 1000 |
| *Dura mater, falx, tentorium* | Ogden 1^st^ order + viscosity  µ_1_=1.78×10^5^ Pa, α_1_=23.07,  G_1_=1.25×10^4^ kPa, G_2_=956 kPa, G_3_=2670 kPa  β_1_=2.37 1/s, β_2_=0.23 1/s, β_3_=0.02 1/s | 1133 |
| *Pia mater* | µ_1_=1.40×10^4^ Pa, α_1_=23.55,  G_1_=1030 kPa, G_2_=78.9 kPa, G_3_=220 kPa  β_1_=2.37 1/s, β_2_=0.23 1/s, β_3_=0.02 1/s | 1133 |
