# Supplementary material for: Performances of the PIPER scalable child human body model in accident reconstruction
Source: PLoS One. 2017 Nov 14;12(11):e0187916. doi: 10.1371/journal.pone.0187916 (PMC5685610; doi:10.1371/journal.pone.0187916)
Supplement: S5 File — (DOCX) [file pone.0187916.s005.docx]

**Supporting Information**

Figure A. **Loading conditions for Case 2012 (left), Case 2017 (middle) and Case 2043 (right).** Linear accelerations of the car B-Pillar during the impact in the physical accident reconstructions were measured by sensors at a sampling frequency of 20,000 Hz. The accelerations in the X, Y and Z directions were filtered by a 6^th^ order low-pass Butterworth filter with a cutoff frequency of 60 Hz which were then applied to the rigid components of the car environment.

**Figure B1.** **Case 2012: linear accelerations in the X, Y and Z directions**. Accelerations of the head (row 1), thorax (row 2), pelvis (row 3) and CRS (row 4) are compared with the Q-dummy measurements. Both the HBM and Q-dummy curves were processed by a low-pass Butterworth filter using the same cutoff frequency of 180 Hz.

**Figure B2.** **Case 2012: upper neck force (row 1), momentum (row 2) in the X, Y, Z directions, and abdomen pressure (row 3)**. Both the HBM and Q-dummy curves were processed by a low-pass Butterworth filter using the same cutoff frequency of 180 Hz.

**Figure C1. Case 2017: linear accelerations in the X, Y and Z directions**. Accelerations of the head (row 1), thorax (row 2), pelvis (row 3) and CRS (row 4) are compared with the Q-dummy measurements. Both the HBM and Q-dummy curves were processed by a low-pass Butterworth filter using the same cutoff frequency of 180 Hz.

**Figure C2.** **Case 2017: upper neck force (row 1), momentum (row 2) in the X, Y, Z directions, and abdomen pressure (row 3)**. Both the HBM and Q-dummy curves were processed by a low-pass Butterworth filter using the same cutoff frequency of 180 Hz.

**Figure D1. Case 2043: linear accelerations in the X, Y and Z directions**. Accelerations of the head (row 1), thorax (row 2), pelvis (row 3) and belt resultant force (row 4) are compared with the Q-dummy measurements. Unlike the other two cases, the data for CRS measurement was not available in the physical reconstruction. Both the HBM and Q-dummy curves were processed by a low-pass Butterworth filter using the same cutoff frequency of 180 Hz.

**Figure D2.** **Case 2043: upper neck force (row 1), momentum (row 2) in the X, Y, Z directions, and abdomen pressure (row 3)**. Both the HBM and Q-dummy curves were processed by a low-pass Butterworth filter using the same cutoff frequency of 180 Hz.
